# PD-L1 Blockade Attenuated Sepsis-Induced Liver Injury in a Mouse Cecal Ligation and Puncture Model

**DOI:** 10.1155/2013/361501

**Published:** 2013-11-11

**Authors:** Weimin Zhu, Rui Bao, Xiaohua Fan, Tianzhu Tao, Jiali Zhu, Jiafeng Wang, Jinbao Li, Lulong Bo, Xiaoming Deng

**Affiliations:** Department of Anesthesiology, Changhai Hospital, Second Military Medical University, 168 Changhai Road, Shanghai 200433, China

## Abstract

Liver plays a major role in hypermetabolism and produces acute phase proteins during systemic inflammatory response syndrome and it is of vital importance in host defense and bacteria clearance. Our previous studies indicated that programmed death-1 (PD-1) and its ligand programmed death ligand-1 (PD-L1) are crucial modulators of host immune responses during sepsis. Our current study was designed to investigate the role of PD-L1 in sepsis-induced liver injury by a mouse cecal ligation and puncture (CLP) model. Our results indicated that there was a significant increase of PD-L1 expression in liver after CLP challenge compared to sham-operated controls, in terms of levels of mRNA transcription and immunohistochemistry. Anti-PD-L1 antibody significantly alleviated the morphology of liver injury in CLP mice. Anti-PD-L1 antibody administration decreased ALT and AST release in CLP mice, decreased the levels of tumor necrosis factor (TNF)-**α**, interleukin (IL)-6, and IL-10 mRNA in liver after sepsis challenge. Thus, anti-PD-L1 antibody might have a therapeutic potential in attenuating liver injury in sepsis.

## 1. Introduction

Sepsis remains a major clinical challenge in intensive care units (ICU), characterized by the systemic inflammatory response and multiple organ failure [1]. Liver is considered as the second organ affected in sepsis and liver dysfunction has been known to occur frequently in cases of sepsis [2, 3]. However, sepsis-induced liver injury, as a part link of sepsis, has always been neglected by critical care physicians [4].

It has been proved that the high rates of morbidity and mortality associated with sepsis may be contributed by immunosuppression caused by impaired pathogen clearance after infection [5–7]. It is accepted that liver plays a major role in hypermetabolism and produces acute phase proteins during systemic inflammatory response syndrome [8]. Changes in hepatic metabolism occur, and they might be of vital importance in host defense and bacteria clearance [9]. In contrast, inflammatory cytokines released during the course of sepsis may also affect metabolic pathways in hepatocytes [10]. Studies designed to investigate the underlying mechanisms and potential therapeutic approaches for sepsis and related organ injury are warranted. 

Programmed death-1 (PD-1) is a newly identified coinhibitory receptor, which has been proved to be an important modulator of immune responses [11, 12]. PD-1 is primarily expressed on the cell surface of activated CD4 and CD8 TCELS. PD-L1 (B7-H1) and PD-L2 (B7-DC) are the two main ligands of PD-1. PD-L1 is broadly expressed on hematopoietic and nonhematopoietic cells, including T cells, B cells, dendritic cells (DCs), macrophages, endothelial cells, epithelial cells, pancreatic islet cells, and fibroblastic reticular cells [12]. The PD-1/PD-L1 pathway has been shown to be a crucial modulator of host immune responses in the regulation of autoimmunity, tumor immunity, transplantation immunity, allergy, immune privilege, and ischemia/reperfusion injury [13, 14]. PD-1/PD-L1 pathway is suggested to play a key role in resisting immune responses of the interaction between host and pathogenic such as certain bacteria [15].

Our previous work showed that the expression of PD-1 and PD-L1 was evaluated in septic patients [16]. We examined the role of PD-1 and PD-L1 in 19 septic shock patients and 22 sex-matched and age-matched healthy controls. We found that compared with healthy controls, septic shock induced a marked increase in apoptosis as detected by the annexin-V binding and active caspase-3 on CD4^+^ T cells, CD8^+^ T cells, and CD19^+^ B cells. Expression of PD-1 on T cells and of PD-L1 on monocytes was dramatically upregulated in septic shock patients. PD-1/PD-L1 pathway blockade *in vitro* with anti-PD-L1 antibody decreased apoptosis of T cells induced by TNF*α* or T-cell receptor ligation. Meanwhile, this blockade potentiated the lipopolysaccharide-induced TNF*α* and IL-6 production and decreased IL-10 production by monocytes *in vitro*. In short, the PD-1/PD-L1 pathway might play an essential role in sepsis-induced immunosuppression. Our animal research found that PD-L1 blockade improved the survival of septic mice [17]. The expression of PD-1 on T cells, B cells, and monocytes and PD-L1 on B cells and monocytes were upregulated in septic animals compared to sham-operated controls. Anti-PD-L1 antibody administration prevented sepsis-induced depletion of lymphocytes, increased tumor necrosis factor (TNF)-*α* and interleukin (IL)-6 productions, decreased IL-10 production, and enhanced bacterial clearance. Anti-PD-L1 antibody administration may be a promising therapeutic strategy for sepsis-induced immunosuppression.

Liver played a key role in the immune tolerance of a variety of diseases. However, the effect of PD-L1 blockade with antibodies on sepsis-induced liver injury and its molecular mechanism remains unclear. Thus, our current research was designed to investigate the role of PD-L1 in sepsis-induced liver injury by a mouse cecal ligation and puncture model. We want to determine the expression of PD-L1 in liver during sepsis and provide a preliminary result of the role of PD-L1 in sepsis-induced liver injury.

## 2. Materials and Methods

### 2.1. Mice

Male 8- to 10-week-old C57BL/6 mice, weighing 22 g to 30 g each, were purchased from the Animals Experimentation Center of Second Military Medical University. All mice were housed in air-filtered, temperature controlled units with 12-hour light-dark cycles and had free access to food and water. All experiments were approved by the Institutional Animal Care and Use Committee of our university.

### 2.2. Induction of Sepsis by CLP

CLP-induced polymicrobial sepsis was performed as described previously [18]. Briefly, mice were anesthetized with isofluorane and a midline abdominal incision was made. The cecum was mobilized, ligated below the ileocecal valve, and punctured twice with a 22 gauge needle to induce polymicrobial peritonitis. The abdominal wall was closed in two layers. Sham-operated mice underwent the same procedure, including opening the peritoneum and exposing the bowel, but without ligation and needle perforation of the cecum. After surgery, the mice were injected with 1 mL physiologic saline solution for fluid resuscitation. All mice had unlimited access to food and water both pre- and postoperatively. A dose of 50 *μ*g anti-PD-L1 antibody (Anti-Mice CD274 (B7-H1) PE, Clone: MIH5, purchased from R&D Systems) in 200 *μ*L of PBS was injected intraperitoneally 1 hour after the operation per mouse. Mouse from the isotype group was injected with a dose of 50 *μ*g (200 *μ*L) control antibody (Rat IgG2a, *λ*) accordingly.

### 2.3. Plasma Biochemistry

Mice were euthanized 24 hours after CLP or sham-operated surgery. Blood samples were taken from the heart. After separation from whole blood by centrifugation, plasma were used for the determination of alanine aminotransferase (ALT) and aspirate aminotransferase (AST) activities by commercially available ELISA kits (Nanjing Jiancheng Bioengineering Institute, Nanjing, China) according to the manufacturer's instructions. 

### 2.4. Hepatic Histology

The right lobe of liver was rapidly harvested after blood obtaining. The liver was then fixed in 10% formalin and then paraffin embedded. 5 *μ*m tissue sections were cut from the paraffin blocks and stained with hematoxylin and eosin. The tissue sections were examined under light microscopy by a senior independent pathologist.

### 2.5. Hepatic Immunohistochemistry

The left lobe of liver was also cut off to be frozen by microtome-cryostat. Consecutive 4-micron sections of the specimens were air-dried for 10 min and then fixed in acetone for 10 min. Endogenous peroxidase activity was blocked by treatment with 0.3% hydrogen peroxidase in PBS for 30 min at room temperature. Sections were then washed three times in PBS. After blocking nonspecific binding with normal solcoseryl for 20 min at room temperature, sections were incubated in a humidified chamber at 4°C overnight with primary antibodies, Anti-PD-L1 antibody, which were purchased from eBioscience (MIH5, San Diego, CA, USA) and used at the final concentrations of 20 *μ*g/mL. Secondary antibodies were used in the morning to bind with primary antibodies after PBS washing and sections were incubated in room temperature for 20 min. The secondary antibodies were detected with DAB according to the manufacturer's instructions, and sections were counterstained with hematoxylin. Negative controls without primary antibody and positive control tissue were included in all experiments to ensure the staining quality.

### 2.6. Hepatic Gene Expression

To evaluate the gene expression of mediators in the inflammatory responses, the mRNA levels of PD-L1, IL-6, IL-10, and TNF-*α* were measured by real-time polymerase chain reaction (RT-PCR). Small cubes of liver were obtained immediately after the death of mice. Total RNA in the cube was extracted using RNeasy Mini kit (Qiagen, Hilden, Germany). 100 ng RNA was used for cDNA synthesis using a High Capacity cDNA Reverse Transcription Kit (Applied Biosystems) according to the manufacturer's protocol. Quantitative RT-PCR was performed using SYBR Green (TaKaRa) on an ABI PRISM 7900 Sequence Detector (Applied Biosystems, USA) with SDS 2.1 software. Each reaction was performed in quadruplicate, with final calculations resulting from means of quadruplicate wells. The ΔΔCq method was used to determine the difference of the mean expression levels of PD-L1, IL-6, IL-10, and TNF-*α* between study subjects with different genotypes of rs4755453. For each individual, the relative expression level ΔCq (Cq T − Cq E) of PD-L1, IL-6, IL-10, and TNF-*α* was normalized with GAPDH and then transformed into relative quantity using the RQ formula (RQ = 2-ΔΔCq, where ΔΔCq is for the individual and ΔCq is the calibrator). The primers for PD-L1 were forward 5′-tgctgcataatcagctacgg-3′ and reverse 5′-gctggtcacattgagaagca-3′. The primers for IL-6 were forward 5′-atggatgctaccaaactggat-3′ and reverse 5′-tgaaggactctggctttgtct-3′. The primers for IL-10 were 5′-ccagttttacctggtagaagtgatg-3′ and reverse 3′-tgtctaggtcctggagtccagcagactc-5′. The primers for TNF-*α* were 5′-catcttctcaaaattcgagtgacaa-3′ and reverse 5′-tgggagtagacaaggtacaaccc-3′.

### 2.7. Statistical Analysis

All data were analyzed using GraphPad Prism software 5.0.1 (GraphPad Software, San Diego, CA, USA). Means and standard errors of the means were calculated in experiments. Paired *t* tests were done when 2 groups were compared. Graphs are displayed as mean with error bars representing the standard error. A *P* value < 0.05 (two-tailed) was considered statistically significant.

## 3. Results

### 3.1. Sepsis Induces the Upregulation of PD-L1 Expression in Liver of Mice

**Figure 1:**
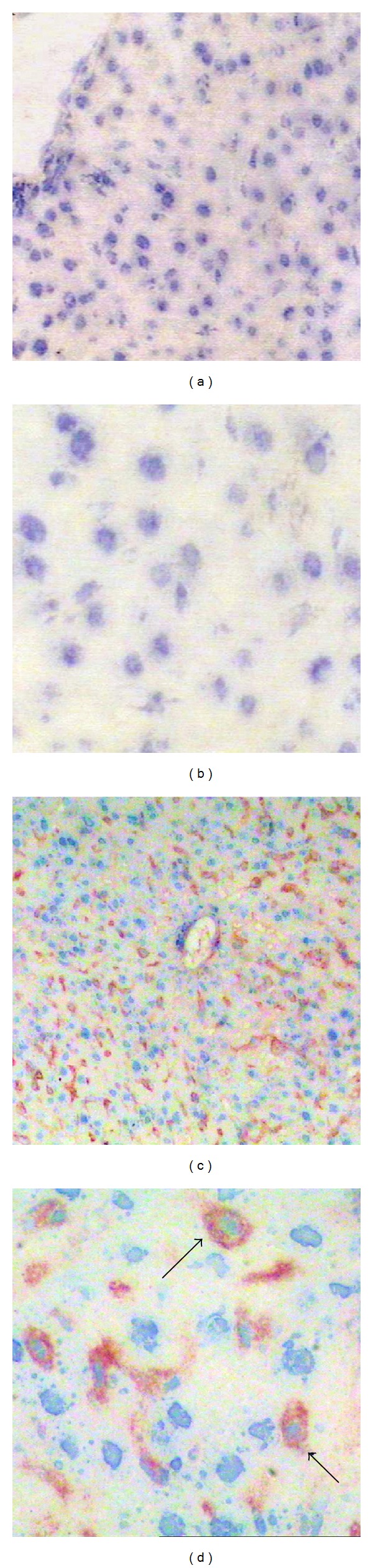
Immunohistochemical staining of liver tissues using anti-PD-L1 Abs. Representative patterns of expression of PD-L1 in liver tissues were shown in sham ((a) and (b)) and CLP mice ((c) and (d)). Positive cells were stained brown. The black arrows indicated PD-L1^+^ cells. Strong and widespread PD-L1 expression was examined as shown in panel (c) and (d), whereas sham groups showed undetectable signal. Original magnification ×200 for (a) and (c) and ×400 for (b) and (d).

To examine the localization of PD-L1 expressions in liver tissues, we performed immunohistochemical staining (Figure 1). Among all specimens from the sepsis group, PD-1 was positively stained on the cell membrane, cytoplasm, or both in a scattered pattern around central vein. There was a wide and strong expression of PD-L1 in the liver of mice after sepsis challenge. On the other hand, there was no staining for PD-L1 activity in the cytoplasm of hepatocytes or other cells of the sham group. 

**Figure 2:**
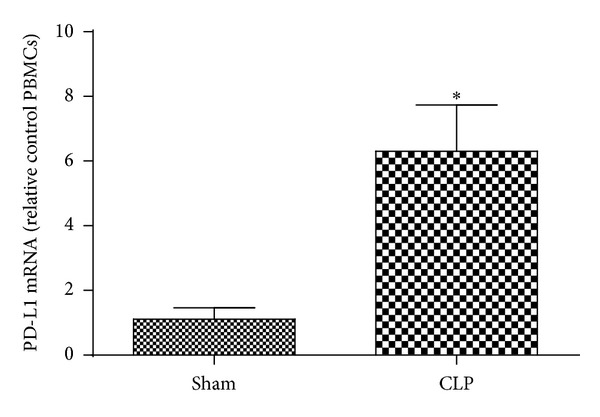
PD-L1 mRNA was unregulated liver of septic mice livers versus Sham at 24 hours. *n* = 8 per each group. *P < 0.01 when mice with CLP were compared with mice from sham group. Bars represent the mean ± SD.

In order to clarify the expression of PD-L1 in liver quantitatively, we performed real-time PCR. Our results indicated that there is a ~5.7-fold significant increase of PD-L1 mRNA expression in liver of mice 24 hours after CLP challenge than that in sham group (Figure 2) (*P* < 0.01). 

These results, based on mRNA transcription and protein expression, reveal an essential role of PD-L1 in liver of mice challenged by CLP-induced polymicrobial sepsis. 

### 3.2. Anti-PD-L1 Antibody Alleviates Morphology of Liver Injury Induced by Sepsis

**Figure 3:**
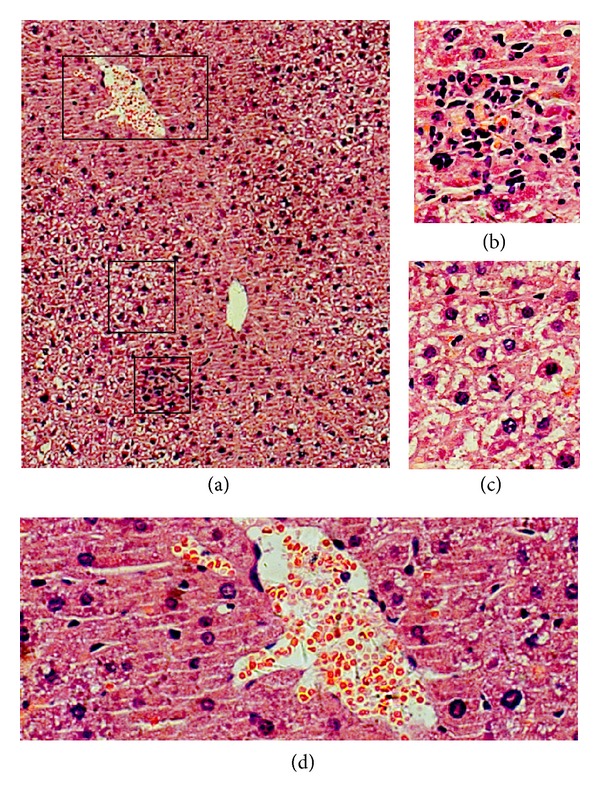
Morphological changes in liver 24 hours after CLP-induced sepsis in hematoxylin and eosin-stained section. Black squares in (a) were magnified into (b), (c), and (d). Spotty necrosis and infiltrations of inflammatory cells were observed in (b). Hepatocytes undergoing ballooning degeneration were observed in (c). Irregular central vein congestion were seen in (d). Original magnification was ×200 for (a) and ×400 for (b), (c), and (d).

H and E staining showed that liver histopathology in mice of sham group was almost normal. In contrast, CLP-induced sepsis led to a significant liver damage in mice at 24 hours. Morphological changes in liver suggested that a systemic inflammation exists (Figure 3(a)). Central vein obstruction was a conspicuous feature in the sepsis group. (Figure 3(a)) Spotty necrosis along with infiltration of mononuclear inflammatory cells exists, which was also noted in the intrasinusoidal area (Figure 3(b)). Hepatocyte hypertrophy and sinusoidal constriction were also found. Remarkably, numerous hepatocytes were undergoing ballooning degeneration (Figure 3(c)). The central vein showed irregularity (Figure 3(d)) and the portal vein congestion could also be observed. On the contrary, mice from the sham group presented normal histology, with normal hepatocytes, portal areas, and parenchyma (not provided).

**Figure 4:**
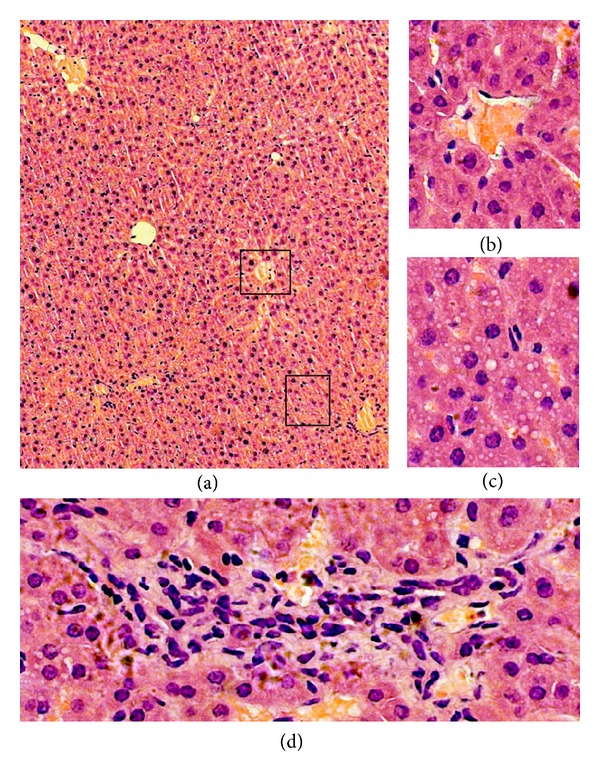
Anti-PD-L1 antibody reverses morphology of liver injury induced by sepsis. Mice were administered anti-PD-L1 antibody (50 *μ*g, i.p.). Liver specimens were sampled 24 hours after surgery. The liver tissue sections were stained with H and E. Black squares in (a) were magnified into (b), (c), and (d). Original magnification was ×200 for (a); ×400 for (b), (c), and (d).

Liver injury was obviously relieved in the anti-PD-L1 group compared with that in the sepsis group under microscope (Figure 4(a)). Although Irregularity could be found in the central vein, spotty necrosis was not significant in the intrasinusoidal area (Figure 4(b)). Fatty degeneration was observed in some hepatocytes. However, the hepatocytes were still normal and the sinusoids were clear (Figure 4(c)). Infiltration of inflammatory cells was only seen in limited plates, which might be the boundary of liver lobules including the portal areas (Figure 4(d)). Thus, PD-L1 blockade by antibodies could reverse the liver injury in mice after sepsis challenge at 24 hours.

### 3.3. Anti-PD-L1 Antibody Decreases ALT and AST Release after Liver Injury Induced by Sepsis

**Figure 5:**
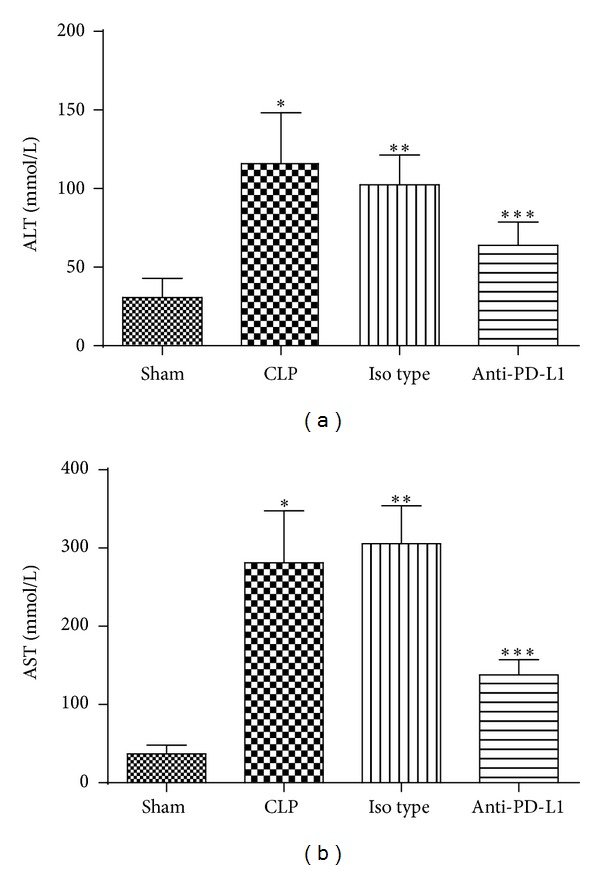
Anti-PD-L1 antibody treatment improved liver function of septic mice. *n* = 8 per each group. *P < 0.01 when mice from CLP group were compared with mice from Sham group; ***P < 0.01 when mice from Anti-PD-L1 group were compared with mice from CLP group; **P > 0.05 when mice from Iso-type group were compared with mice from CLP group. Bars represent the mean ± SD.

As expected, CLP-induced sepsis caused increased levels of ALT and AST in mice (115.88 ± 32.30 mmol/L and 281.13 ± 66.43 mmol/L, resp.), indicating liver damage, compared with sham group (30.75 ± 12.14 mmol/L and 36.75 ± 11.39 mmol/L, resp.) (*P* < 0.01). In contrast, anti-PD-L1 antibody decreased levels of ALT and AST (63.88 ± 14.83 mmol/L and 137.75 ± 19.55 mmol/L, resp.) (*P* < 0.01). Thus, anti-PD-L1 antibody could reduce the level of ALT and AST levels (Figure 5). 

### 3.4. Effect of PD-L1 Blockade on the Level of IL-6, IL-10, and TNF-*α* mRNA in Liver

**Figure 6:**
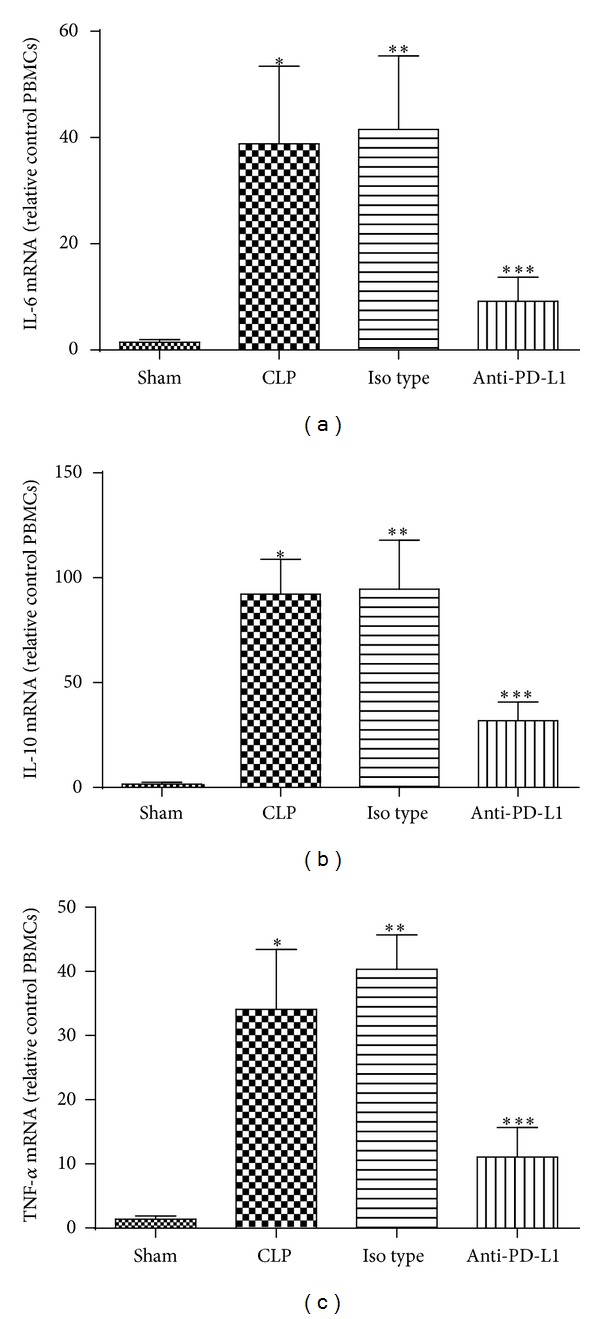
Anti-PD-L1 antibody altered mRNA expression of IL-6, IL-10, and TNF-*α* in the liver of septic mice. Mice were administered anti-PD-L1 antibody (50 *μ*g, i.p.). Liver specimens were sampled 24 hours after surgery. *P < 0.01 versus Sham, ***P < 0.01 versus CLP, **P = 0.7128 (IL-6), *P* = 0.8133 (IL-10), and *P* = 0.1228 (TNF-*α*) versus CLP. Bars represent the mean ± SD of eight mice for each group.

We then evaluated the therapeutic effect of anti-PD-L1 antibody on the mRNA levels of several cytokines at 24 hours after sepsis. The mRNA levels of IL-6, IL-10, and TNF-*α* were all increased in CLP group compared to sham group. Anti-PD-L1 antibody treatment significantly reduced the mRNA levels of IL-6, IL-10, and TNF-*α* in the liver (*P* < 0.01). There is no significant difference between CLP group and Isotype group in terms of each cytokine (*P* > 0.05) (Figure 6). These results indicated that therapeutic administration of anti-PD-L1 antibody relieved the inflammation caused by CLP-induced sepsis in liver. 

## 4. Discussion

The main finding of this study is that PD-L1 expression was regulated in the liver of mice with sepsis, based on levels of mRNA transcription and Immunohistochemistry of liver. PD-L1 blockade with antibodies could decrease the level of CLP-induced septic liver injury.

CLP-induced sepsis caused a significant inflammatory response and damage in a liver characterized both biochemically (ALT and AST activity) and histologically (hematoxylin-eosin-stained section). Our findings are in accordance with the report by Koskinas et al. [19]. They showed that patients dying from sepsis were mainly associated with portal inflammation, centrilobular necrosis, and hepatocellular apoptosis. Increased levels of AST and ALT were observed in patients dying from sepsis [19].

Sepsis was associated with overproductions of proinflammatory cytokines, which can lead to the recruitment of leukocytes and tissue damage [20, 21]. A number of inflammatory cytokines, including IL-6, IL-10, IL-1, and TNF-*α* were involved in sepsis, which contributes to tissue and organ damage. Our study found that anti-PD-L1 antibody suppressed not only proinflammatory cytokines (IL-6 and TNF-*α*) but also anti-inflammatory cytokine (IL-10). These results are partially in accordance with our previous findings. Zhang et al. [17] found that PD-L1 blockade significantly increased the expression of TNF-*α* and Il-6 and decreased the level of IL-10 in CLP murine plasma. Collectively, these data indicated that the protective effects of anti-PD-L1 antibody on septic liver injury might be partially achieved by inhibition of proinflammatory cytokines.

With the use of CLP-induced sepsis, a well-established animal model for sepsis, our findings indicated that PD-L1 expression was regulated in the liver of mice with sepsis. Mice with sepsis had a significant elevation of PD-L1 expression in liver, based on the levels of mRNA and transcription immunohistochemistry staining of liver. It was indicated that PD-L1 was expressed on Kupffer cells (KCs) and liver sinusoidal epithelial cells in murine liver tissues (LSECs) [22]. Hepatocytes express constitutively low levels of PD-L1 but show an increase of PD-L1 expression upon stimulation with interferons [23]. Further studies were needed to clarify the exact localization of PD-L1 by immunofluorescence double staining or flow cytometry in liver specimens. Based on the current understanding of the role of PD-L1 in liver, we speculate that upregulation of PD-L1 was mainly on KCs and infiltrated lymphocytes. Whether sepsis may alter the expression of PD-1 and PD-L2 in liver also needs to be investigated.

Several limitations in our study should be noted. We did not explore more specific mechanisms underlying the protective role of anti-PD-L1 antibody in sepsis-induced liver injury. It is reported that liver damage during sepsis is manifested by functional change rather than structure damage. A time course study of the role of PD-L1 in sepsis-induced liver injury is warranted. Our current research chose a single dose of anti-PD-L1 antibody to use on the mice, which was based on our previous findings. The effects of anti-PD-L1 antibody at different doses in the model also need to be clarified. Furthermore, whether the blockade of PD-L1 plays a vital role in other organ damages, that is, kidney, intestinal, and heart, during sepsis also needs to be clarified.

## 5. Conclusion

To conclude, we found elevated PD-L1 expression in liver of mice with experimental sepsis. PD-L1 blockade with antibodies could exert protective roles against sepsis-induced liver injury, characterized by alleviating liver pathological injury, reducing ALT and AST release, and attenuating liver inflammatory responses. Thus, anti-PD-L1 antibody might have a therapeutic potential in reducing liver injury in sepsis.
